# Disease expression caused by different variants in the *BEST1* gene: genotype and phenotype findings in bestrophinopathies

**DOI:** 10.1111/aos.14958

**Published:** 2021-07-29

**Authors:** Katarzyna Nowomiejska, Fadi Nasser, Katarina Stingl, Simone Schimpf-Linzenbold, Saskia Biskup, Agnieszka Brzozowska, Robert Rejdak, Susanne Kohl, Eberhart Zrenner

**Affiliations:** 1 Chair and Department of General and Pediatric Ophthalmology, Medical University of Lublin, Lublin, Poland; 2 Institute for Ophthalmic Research, Center for Ophthalmology, University of Tübingen, Tübingen, Germany; 3 University Eye Hospital, Center for Ophthalmology, University of Tübingen, Tübingen, Germany; 4 Center for Rare Eye Diseases, University of Tübingen, Tübingen, Germany; 5 Praxis für Humangenetik Tübingen, Tübingen, Germany; 6 Department of Mathematics and Medical Biostatistics, Medical University of Lublin, Lublin, Poland; 7 Werner Reichardt Centre for Integrative Neuroscience, University of Tubingen, Tübingen, Germany

**Keywords:** autosomal dominant bestrophinopathy, best vitelliform macular dystrophy, genotype, phenotype

## Abstract

**Purpose:**

To analyse the spectrum of clinical features and molecular genetic data in a series of patients carrying likely disease-associated variants in the *BEST1* gene.

**Methods:**

Retrospective observational analysis of clinical data extracted from the medical records of visual function, multimodal imaging and electrophysiology of 62 eyes of 31 patients. Molecular genetic analysis was performed by means of panel-based NGS or Sanger sequencing.

**Results:**

The spectrum of variants in the *BEST1* gene comprised 19 different variants and three of which are novel. Fundus photographs and OCT images allowed categorization of 52 eyes as Best vitelliform macular dystrophy (BVMD) with stages 1 to 5 and 10 eyes with autosomal recessive bestrophinopathy (ARB), with more severe phenotype. One patient was shown to be heterozygous for a variant, which has so far been described only in ARB, but this patient had the BVMD phenotype. There was no significant progression of the visual acuity during the follow-up period of 5 years both in BVMD and ARB. The most prevalent pattern of fundus autofluorescence (FAF) in BVMD was ‘patchy’. There were diverse visual field defects in static automated perimetry (SAP) depending on the stage. The Arden ratio was significantly lower in ARB patients and in eyes with stage 5 of BVMD.

**Conclusions:**

The genotype does not always predict the phenotype in patients with BVMD and ARB; however, having two mutations in the *BEST1* gene causes a more severephenotype. FAFhelped to distinguish ARB from BVMD. Most of the observed eyesdidnotprogressfunctionallyduringthefollow-up.ARBandtheatrophicstageof BVMD as the disease end-stage had the worst visual functions and EOG results.

## Introduction

The *BEST1* gene (MIM607854) is located on the long arm of chromosome 11 (11q13) (Marmorstein et al. 2000). The *BEST1* gene encodes a protein named bestrophin-1, which is a Ca^2+^-sensitive pentameric chloride channel located on the basolateral membrane of retinal pigment epithelial (RPE) cells (Boon et al. 2009). Bestrophin-1 is responsible for regulation of the transepithelial ion transport of intracellular calcium signalling and RPE cell volume and for modulation of homeostasis in the subretinal space (Hartzell et al. 2008). Although the precise role of the *BEST1* protein has not yet been fully elucidated, it is known that mutations in the *BEST1* gene lead to abnormal function of the RPE and outer photoreceptor segments and central neurosensory retinal detachment (Frangieh et al. 1982).

Over 300 mutations in the *BEST1* gene have been identified and published so far in humans (Burgess et al. 2008; Pasquay et al. 2015; Tian et al. 2017) that are associated with the following spectrum of retinal disorders summarized as ‘bestrophinopathies’: vitelliform macular dystrophy 1 (VMD1, MIM153840), Best vitelliform macular dystrophy (BVMD, VMD2, MIM153700), autosomal recessive bestrophinopathy (ARB, MIM611809), vitreoretinochoroidopathy (VRCP, MIM193220) and retinitis pigmentosa 50 (RP50; MIM613194).

The most common bestrophinopathy is BVMD, that is an autosomal dominant condition with a prevalence of 1:50 000 (Bitner et al. 2012). It is associated with a reduced Arden ratio in the electro-oculogram (EOG) (Arden & Constable 2006) and an accumulation of yellowish material between the RPE and photoreceptors in the macula (Marmorstein et al. 2000; Boon et al. 2009). Bilateral vitelliform lesions evolve over time through five progressive stages: previtelliform, vitelliform, pseudohypopyon, vitelliruptive and atrophic macular lesions^10^. Typically, patients start with central visual loss in the first or second decade of life, and later there is progression of the visual acuity loss due to disintegration of the yellowish lesions and following atrophy.

ARB caused by recessive mutations in the *BEST1* gene is a distinct phenotype, which was first reported in 2008 (Burgess et al. 2008). The estimated prevalence of ARB is about 1: 1 000 000 (Milenkovic et al. 2018). Clinically, there are no typical central vitelliform lesions in ARB, but widespread irregularity of the RPE with abnormal fundus autofluorescence (FAF) and accumulation of subretinal fluid or macular oedema are detected (Burgess et al. 2008). Other phenotypes associated with *BEST1* mutations are less common. It is still not known how different mutations in *BEST1* lead to clinically distinct retinopathies.

The aim of this study was to explore genotype and phenotype features in a group of patients with confirmed likely pathogenic variants in the *BEST1* gene, examined with multimodal imaging in a longitudinal follow-up.

## Materials and Methods

The study was designed as a retrospective observational case series. It was conducted at the Clinic for Hereditary Retinal Dystrophies at the Center for Ophthalmology of the University of Tübingen in Germany. The study was performed in accordance with the tenets of the Declaration of Helsinki established in 1975 (revised in 1983) and was approved by the Ethics Committee at the Eberhard Karls University, Tübingen, Germany (approval number 126/2018BO2).

### Patients and clinical examinations

The medical records of 62 eyes of 31 patients (17 males, 14 females) with clinically and genetically confirmed likely pathogenic *BEST1* variants were analysed retrospectively. Extensive medical and family history was taken from each patient. All patients underwent best-corrected visual acuity (BCVA, Snellen) testing, fundus examination, optical coherence tomography (OCT, HRA-OCT; Heidelberg Engineering, Heidelberg, Germany) and short-wavelength fundus autofluorescence imaging (FAF, HRA 2; Heidelberg Engineering, Heidelberg, Germany). Additionally, full-field electroretinograms (ERG), multifocal ERGs (mfERG) and electrooculograms (EOG) were recorded (Espion Diagnosys LLC, Lowell, MA, USA) in accordance with the standards of the International Society for Clinical Electrophysiology of Vision (ISCEV) (McCulloch et al. 2015).

Visual field examinations were performed with the 30-degree suprathreshold strategy of static automated perimetry (SAP, Octopus 900, Haag-Streit, Koenitz, Switzerland), using the Goldmann white III stimulus presented for 200 mseconds on a background of 10 cd/m^2^.

### Molecular genetic assessment

All patients provided blood samples for genetic testing. The patients were screened using either a custom capture panel targeting 105 retinal disease genes (Weisschuh et al. 2020) or conventional Sanger sequencing of the coding exons of the *BEST1* gene. Validation of called variants upon next-generation sequencing was performed by means of bidirectional Sanger sequencing. If possible, variants were also tested for co-segregation within kinships.

### Data analysis

The BCVA was expressed in decimals and converted to logMAR. Values of refraction were converted to a spherical equivalent.

BVMD and ARB were diagnosed on the basis of fundus photographs and FAF images. The stage of BVMD was classified from the fundus photographs according to the Gass classification (Gass 1997) as follows: stage 1 – previtelliform (subclinical, with normal or almost normal appearance of the fovea), stage 2 – central or paracentral vitelliform lesion, stage 3 – pseudohypopyon, stage 4 – vitelliruptive and stage 5 – atrophic. The FAF images were classified according to the Parodi classification (Parodi et al. 2014a): normal pattern (no difference in FAF appearance compared with a normal subject), hyper-autofluorescent pattern (increased FAF signal), hypoautofluorescent pattern (decreased FAF signal), patchy pattern (combined increased and decreased FAF signal), multifocal pattern (multiple, isolated increased FAF signals) and spoke-like pattern (increased FAF signals with a spoke-like configuration). Additionally, the presence of a new feature in FAF, that is peripapillary sparing, characterized by the absence of flecks and RPE atrophy in the peripapillary region of the retina, was analysed (Birtel et al. 2020).

Vertical high-resolution OCT line scans of the fovea were selected for analysis, and the central retinal thickness was measured in micrometres (μm) with the calliper tool provided by the software of the device.

The visual field results were classified as a normal relative central scotoma, an absolute central scotoma and a diffuse scotoma. To assess the results quantitatively, the number of relative and absolute defects was taken into consideration. A relative defect was defined as one where the standard test object was not seen, but there was a response to brighter stimulus, whereas an absolute defect was defined as one where the stimulus was not seen at any luminance, even at the maximal luminosity.

An Arden ratio (the ratio of the light peak/dark trough) less than 1.5 was considered pathological in the EOG (Arden & Constable 2006). Progression was based on a reduction in the visual acuity and on the FAF and OCT results over time during the mean period of 4.5 ± 4.6 years of the follow-up.

Statistical analysis was performed using STATISTICA 13.0 software (StatSoft, Krakow, Poland). All values were presented as medians. The Kruskal–Wallis test was used to compare independent groups. Bivariate relationships were analysed using the Spearman correlation coefficient comparisons of means. A value of p < 0.05 was considered statistically significant.

## Results

### Patient cohort

The mean age at the onset of visual symptoms, defined as a decrease in the visual acuity, was 26 years (range 10–64 years). The mean age of the patients was 41 years (range: 16–71 years) at the first visit and 45 years (range: 17–76 years) at the last visit. One patient was diagnosed with glaucoma (MB83), one with suspicion of glaucoma (MB48), three patients with cataract in both eyes (MB6, MDS290, MB31) and one with keratoconus (MB80). The mean number of examinations during the follow-up was three (range 1–10) (Table 1).

**Fig. 1.**
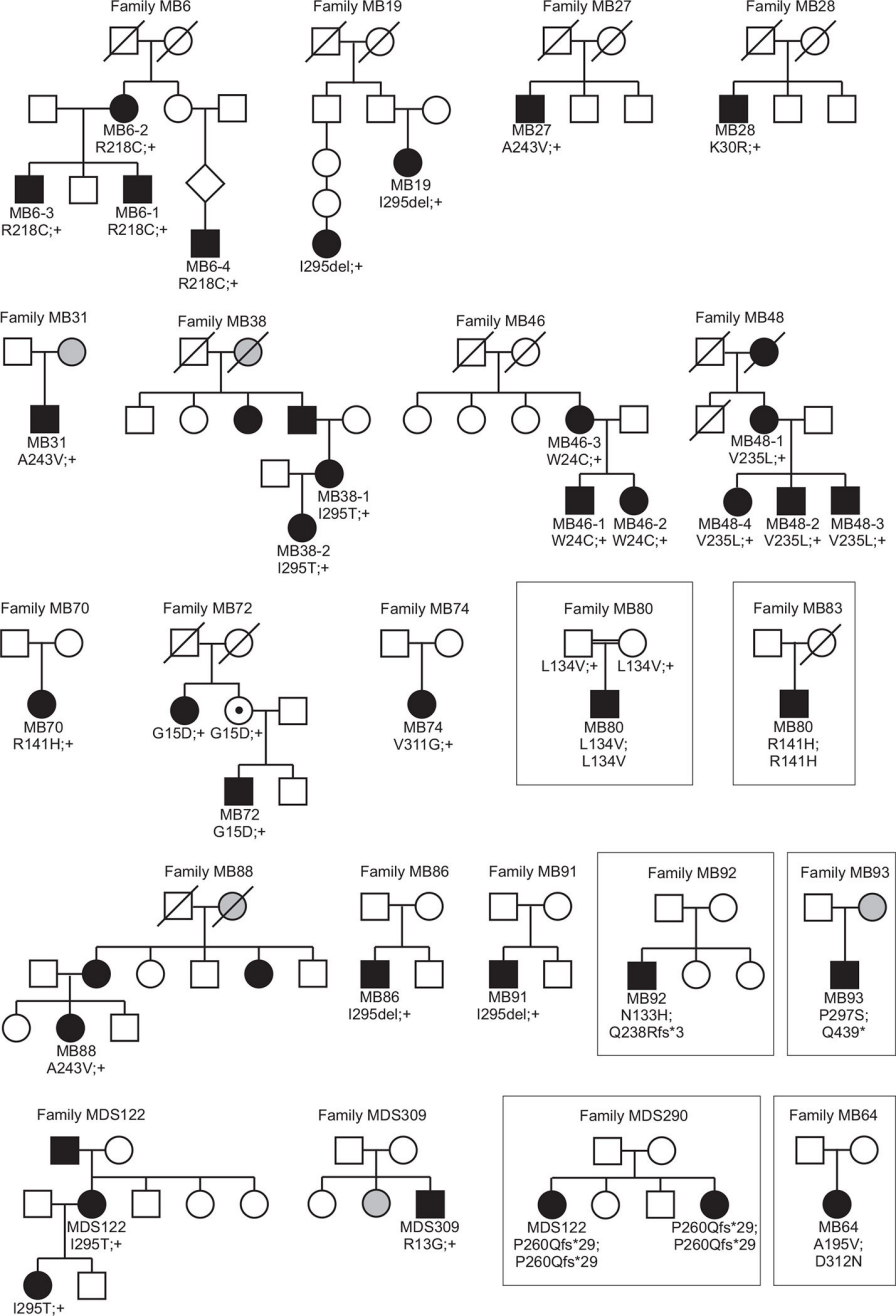
Pedigrees of the patients and families analysed in this study. The patients clinically assessed in this study are indicated by their family ID; if there are multiple individuals within a family, this is followed by an individual identifier. Affected individuals are given by black filled symbols, self-reported/anamnestic affected individuals in grey filled symbols and healthy mutation carriers with a black dot. Note the unaffected carrier in family MB72. Genotypes are given below each available family member by the deduced change at the amino acid level; ‘+’ denotes the wildtype allele. Families/patients with a putative autosomal recessive mode of inheritance are boxed in.

Overall, 31 patients from 22 families were included in this study (see pedigrees in Fig. 1). Thirteen patients were members of four families (MB6 – four patients, MB38 – two patients, MB46 – three patients, MB48 – four patients), six patients had a family history of macular disease, and the other 12 patients were sporadic cases (with unknown family history). Our cohort consisted of families mainly of German ancestry; only two patients (MB80 and MB93) had Arabic origin and two patients (MB72 and MDS 309) were Italian.

### Mutation analysis of the *BEST1* gene

The mutation spectrum comprises 19 different variants, three of which are novel (Table 2).

Missense variants (*n* = 15) were the most frequent mutation type. In addition, we identified one nonsense variant, one in-frame deletion and two frameshift deletions.

In 24 patients/15 families, we suspected a dominant inheritance pattern, as all but one novel variants have already been described to be disease-causing in BVMD. In six patients, we suspected a recessive mode of inheritance: three patients were homozygous for variants that had previously been found in ARB cases, while three patients were found to be heterozygous for two putative disease-causing variants. One patient (MB70) was shown to be heterozygous for the known c.422G>A;p.R141H variant (Krämer et al. 2000). The phenotype of this patient was BVMD with progression from central to multifocal vitelliform lesions. We can exclude that copy number variations (CNVs) in *BEST1* constitute the second allele in this patient, as we performed multiplex ligation-dependent probe amplification and found no deletions or duplications. Whether noncoding deep-intronic variants in the *BEST1* gene account for the second pathogenic allele remains unknown. Of course, we cannot rule out that the phenotype of patient MB70 is not related to this *BEST1* variant.

### Clinical features

Table 3 shows the number of cases obtained after classification of the 62 eyes according to the fundus appearance (Gass classification) and OCT (vertical panels) and after classification based on the autofluorescence (FAF) images (horizontal panels).

#### Fundus photographs

The fundus images of 24 patients (48 eyes) with a dominant mode of inheritance and two eyes with unclear inheritance allowed the following classification: five eyes (8%; patients from families MB74, MDS309) – previtelliform stage 1 according to the Gass classification with no retinal changes, eight eyes (12%; patients from families MB88, MB31, MB28, MB48, MB86) – vitelliform stage 2 with a yellow, well-demarcated, central vitelliform lesion, 6 eyes (10%) (patients from families MB19, MB6, MDS122) – pseudohypopyon stage 3, 14 eyes (23%; patients from families MB46, MB91, MB72, MB38, M48) – vitelliruptive stage 4 and 19 eyes (31%; patients from families MB48, MB6, MB38) – atrophic stage 5 (Table 3).

Asymmetry between both eyes of one patient with respect to Gass stages was observed in six patients: stage 1/stage 2 (MB70), stage 2/stage 4 (MB48 member 3), stage 3/stage 5 (MB6, MDS122) and stage 4/ stage 5 (MB48 – member 2).

Ten eyes with the ARB phenotype and a possible recessive mode of inheritance revealed no central retinal changes (Table S1).

#### Fundus autofluorescence

From the FAF images, we identified 52 eyes with a central lesion – the BVMD phenotype, and ten eyes with a spread appearance of FAF outside the posterior pole – the ARB phenotype (Table 2).

In the group with the ARB phenotype, ten eyes exhibited diffuse irregularities of the RPE, large hypofluorescent areas in the posterior pole including dispersed punctate flecks alterations of the RPE and white subretinal deposits in the macular area and midperiphery (Table 3). Peripapillary sparing was observed in eight of the ten eyes of five patients with the ARB phenotype; it was not present in the case of MDS290. FAF images of the first and last visit of eyes that progressed in loss of visual acuity are presented in Table 3.

#### Optical coherence tomography

There was almost normal appearance of the fovea with the layer between the RPE and the inner segment and outer segment (IS/OS) interface in all five eyes in stage 1 of BVMD (Table 2). In stage 2, a round hyperreflective vitelliform material beneath the retina and RPE was present in all cases, and concomitant detachment of the RPE from the neurosensory retina was observed in five cases. In stage 3, there was subretinal fluid with loss of vitelliform material in all cases. In stage 4, all 14 cases showed central neurosensory retinal detachment with residual lipofuscin. In stage 5, atrophy was found in all cases with a decrease in the area of vitelliform material. Choroidal excavation, described as a cup-shaped focal choroidal excavation in the macula in OCT (Parodi et al. 2014b), was seen in both eyes of one patient. In the eyes of patients with the ARB phenotype, remains of intraretinal fluid were found between the retina and the RPE in five eyes (Table 2). None of the patients exhibited choroidal neovascularization with the need for treatment with intravitreal anti-VEGF injections. The OCT images of eyes that progressed, along with the visual acuity, taken at the first and last visit are presented in Table S2.

**Fig. 2.**
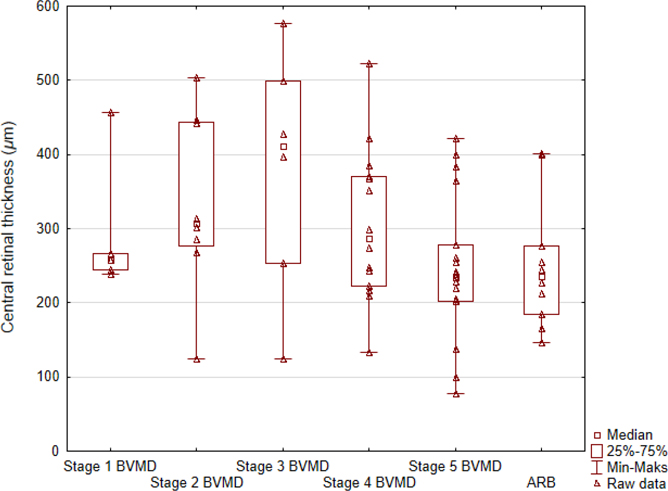
Values of central retinal thickness (μm) measured with the use of optical coherence tomography (OCT) during the first examination of all 62 eyes in five stages (1–5) of Best vitelliform macular dystrophy (BVMD) and autosomal recessive bestrophinopathy (ARB). Each data point means value of central retinal thickness in a single patient.

Figure 2 shows the median central retinal thickness (CRT) at different BVMD stages in ARB patients during the first examination of all 62 eyes. The differences were close to significance (p = 0.06), with the median CRT increasing from stage 1 (261 μm) to stage 3 in BVMD (348 μm) and then decreasing to stage 5 (237 μm). The CRT of eyes from stage 1 to stage 4 was significantly higher (p = 0.01) than that of eyes from stage 5 and ARB. There was no significant correlation between the CRT and BCVA.

#### Refractive error

The majority of eyes were hyperopic (36 eyes). There were 21 eyes with myopia and five emmetropic eyes. Amblyopia was reported in two patients, and keratoconus was diagnosed in one patient (MB80-ARB phenotype). There were no significant differences in the spherical equivalent between the groups.

#### Visual field

**Fig. 3.**
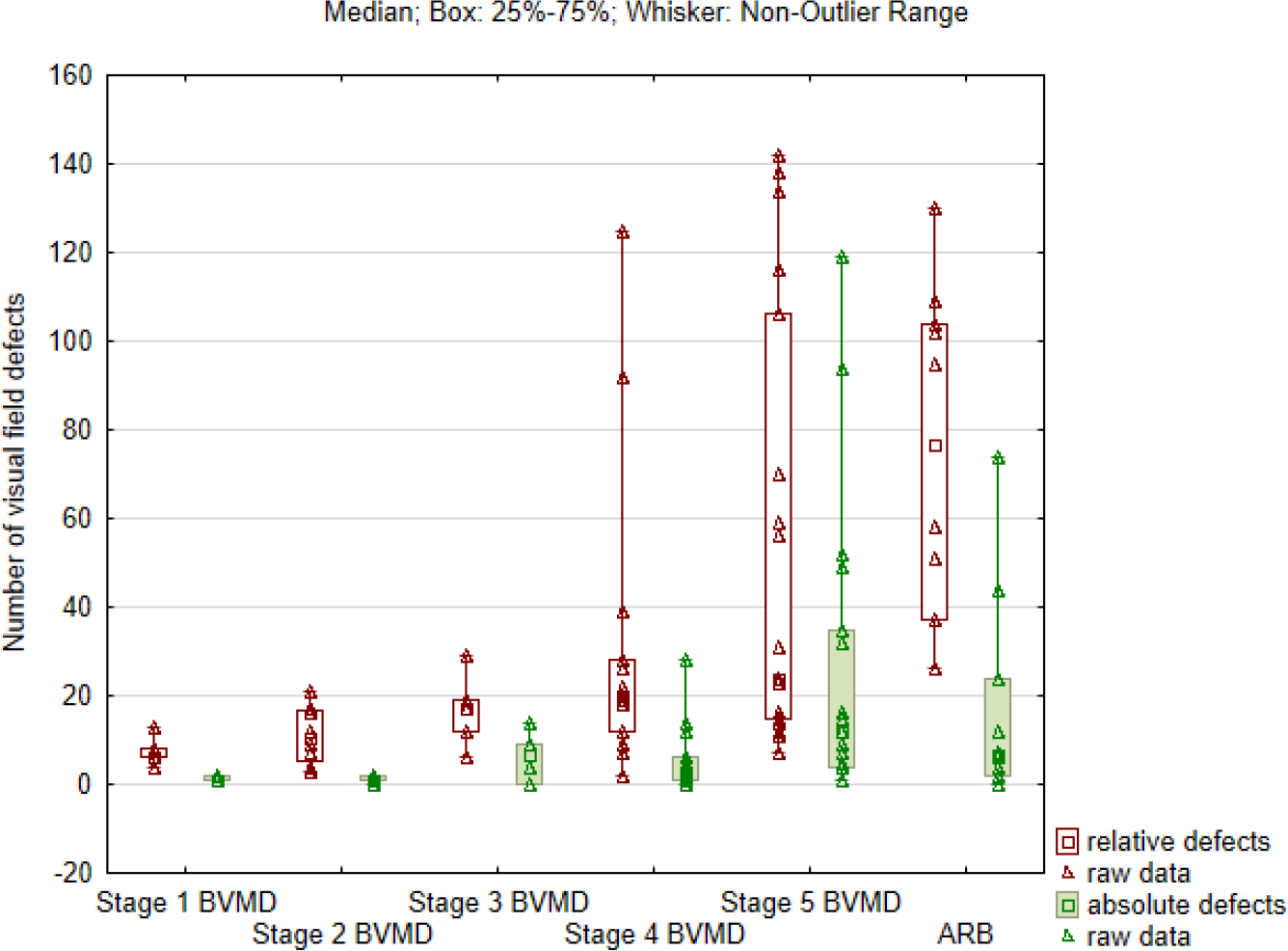
The number of relative (white bars) and absolute (green bars) visual field defects in static automated perimetry within 30-degree eccentricity obtained during the first visit in 50 eyes with five stages (1–5) of Best vitelliform macular dystrophy (BVMD) and autosomal recessive bestrophinopathy (ARB).

Figure 3 presents the median number of relative or absolute visual field defects that were detected within 30-degree eccentricity using SAP. Patients with other comorbidities, such as glaucoma (MB83), suspicion of glaucoma (MB48), cataract (MB6, MDS290, MB31) and keratoconus (MB80), were excluded from this analysis, as these diseases may affect the visual function. Thus, visual field results of 50 eyes were taken into consideration. There was a significant correlation (p = 0.001) between the scotoma type and the stage of the disease. In stages 1–4 of BVMD, a relative central scotoma was the most common finding in the SAP results (45%), whereas an absolute central scotoma was most common in stage 5 (58%; atrophic stage) and a general reduction in sensitivity was noted in 40% of the ARB patients. There were significant differences between the number of relative and absolute scotomata between the BVMD stages(p = 0.0005). The highest number of relative scotoma was found in the ARB group, and the highest number of absolute scotoma was found in stage 5 (atrophic).

#### Electrophysiology

In all patients, the Arden ratio was under 150% in the EOG examination. The mean Arden ratio was significantly higher (p = 0.04) in the eyes with stages 1–4 (132%; range 122–144%) than in the eyes with ARB (127%; range 117–128%) and the atrophic stage of BVMD (120%; range 110–130%). The full-field ERG results in both scotopic and photopic conditions were within the normal range, except for four eyes of three patients with slightly reduced amplitudes (patients MDS290 and MB86 – ARB phenotype, MB83 – BVMD phenotype). The multifocal ERG results were abnormal in the central area for all except three eyes with stage 1 BVMD (patients MB74, MB70), three eyes with stage 2 BVMD (patients MB31, MB70) and two eyes with stage 3 BVMD (patients MB64), which were normal.

#### Visual acuity

There were significant differences in the visual acuity between the BVMD stages and ARB at the first baseline visit (p = 0.0004) and the last visit of the follow-up (p = 0.0001). The worst visual acuity was observed in eyes with atrophic stage of BVMD and ARB phenotype. BCVA progression data were available for 46 eyes. The drop in the visual acuity was reported in 20 eyes, which indicates that 26 eyes did not progress. The visual acuity of eyes that progressed between the first and last visit is presented in Table S2.

**Fig. 4.**
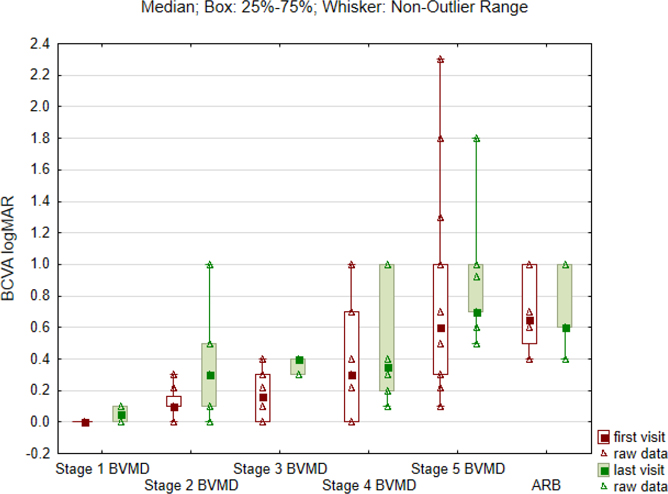
Values of the median best-corrected visual acuity (BCVA) in logMAR obtained at the first and last visit for all 62 eyes along five Gass stages (1–5) of Best vitelliform macular dystrophy (BVMD) and autosomal recessive bestrophinopathy (ARB).

There were no significant differences in the visual acuity between the follow-up visits. Figure 4 shows the median of the BCVA (logMAR) at the first and last visits.

## Discussion

There is a considerable spectrum of phenotypic expression both in BVMD and ARB, as there is variable penetrance and expressivity of the *BEST1* mutation-associated retinal phenotypes (Marmorstein et al. 2009). Little is known about the exact mechanism of reduced penetrance and variable expression, and the causes of the distinct retinal phenotypes are still not fully understood.

Many *BEST1* mutations are rare and found in single families (Schatz et al. 2010; Sodi et al. 2012; Gao et al. 2018), and they have been investigated in several different ethnic groups (Italian, Dutch, Swedish, German, Chinese, Australian) (Deutman 1971; Tian et al. 2017; Gao et al. 2020). The majority of our patients have German origin. Krämer et al. (2000) have also described a group of 41 BVMD patients with German origin in whom they identified 23 distinct disease-causing variants in *BEST1*. Three of the variants observed by the authors were also found in our cohort, that is c.728C>T;p.A243V and c.884_886del; p.I2965del recurrently in three cases each and c.932T>G; V311G in a single patient.

We describe three new likely disease-associated variants: one associated with the BVMD phenotype and two with the ARB phenotype (see Table 2). Almost all mutations (92%) in the *BEST1* gene described so far are missense mutations (Boon et al. 2009), resulting in amino acid changes in the N-terminal part of the protein located within the first 310 residues, often within or close to transmembrane domains (Kinnick et al. 2011; Boon et al. 2013). This is consistent with our results. There is still no explanation as to why the various missense mutations result in distinct clinical phenotypes. Mutations in the *BEST1* gene have pleiotropic effects, and the phenotypes are expected to be influenced by the age, sex, environment and presence of modifier genes (Guziewicz et al. 2018). It is already known that compound heterozygous or homozygous mutations in *BEST1* may confer a particularly severe phenotype of ARB (Burgess et al. 2008; Gao et al. 2018).

Five of six patients with a recessive mode of inheritance from our study exhibited features of ARB. The visual acuity of ARB patients is usually poor, less than 20/60 in both eyes, and the mean age of onset is 23 years (Burgess et al. 2008; Gao et al. 2018). Retinal oedema and neurosensory retinal detachment with subretinal fluid can be observed, and no vitelliform lesions develop^5^. In our group of ARB patients, the intraretinal fluid was present in five of ten eyes that had the worst visual acuity and EOG results.

In ARB, nonsense variants and frameshift deletions lead to mutant transcripts that are most probably targeted to nonsense-mediated decay: these patients may not express bestrophin-1 in the RPE plasma membrane. Among the 270 mutations described for *BEST1* so far (Fokkema et al. 2005), only about 40 compound heterozygous and homozygous mutations have been associated with ARB (Burgess et al. 2008; Schatz et al. 2010).

Patients with ARB are usually compound heterozygous (Burgess et al. 2008) or homozygous carriers (Bitner et al. 2011) of pathogenic *BEST1* mutations, while heterozygous parents generally show no retinal symptoms. In our study, one ARB patient was homozygous for a null allele (MDS290) and two for a missense variant (MB80, MB83). Two ARB patients were probably compound heterozygous for a null and missense allele (MB92 – confirmed, MB93 – suspected). Patient MB64 was heterozygous for two missense variants. One of these patients had the phenotype of BVMD with a pseudohypopyon stage (MB 70). He was shown to be heterozygous for a variant, which has so far been described only in ARB (Krämer et al. 2000), but this patient had the BVMD phenotype. It means that the genotype does not always predict the phenotype.

The phenotype of multifocal vitelliform lesions (severe dominant BVMD) in a patient homozygous for a *BEST1* mutation has already been described in a Danish family (Piñeiro-Gallego et al. 2011).

In BVMD, there is extensive accumulation of lipofuscin within RPE cells, resulting clinically in a yellow subretinal lesion (Marmorstein et al. 2009; Tian et al. 2017) with corresponding choroidal thickening (Parodi et al. 2016b). It has been shown that, phenotypically, the main clinical fundoscopic findings in BVMD are localized in the posterior pole (Boon et al. 2009). Using FAF imaging (Lima de Carvalho et al. 2019), retinal regions away from the central lesion in BVMD tend to appear normal; thus, RPE lipofuscin levels are not increased. Central vitelliform lesions demonstrate increased FAF *in vivo*, suggesting that the yellow pigment contains lipofuscin (Miller, 1978) – waste products of the visual cycle, such as A2E^34^. It is probable that the intense FAF within the lesion indicates that the rate of bisretinoid production in the outer segments of photoreceptor cells is accelerated within the lesion due to the impairment of photoreceptor cells located in the fluid-filled lesion (Parodi et al. 2016a). There are also histopathological studies confirming an increase in lipofuscin in the RPE of donor eyes (Bakall et al. 2007). In the vitelliruptive stage of BVMD, there is disintegration of the lipofuscin material and the presence of the subretinal fluid, which might be misdiagnosed with the central serous chorioretinopathy, if there is asymmetry between eyes in BVMD. The final atrophic stage of BVMD with the fibrotic tissue might be similar to more common macular dystrophy – Stargardt disease or advanced stage of bilateral age-related macular degeneration (AMD). In ARB, there are no vitelliform lesions; however, bilateral macular oedema may be present which can mimic macular oedema of different origin, for example diabetic macular oedema or oedema secondary to retinal vein occlusion. Moreover, thin separation of the photoreceptors from the RPE seen in OCT in eyes with ARB as a result of accumulation of the fluid may be confused with the wet form of AMD. Thus, clinically suspected cases of ARB warrant genetic testing to confirm the diagnosis (Boon et al. 2013).

FAF facilitates visualization of the distribution of A2E and other bisretinoid pigments of lipofuscin material in the RPE; thus, it can contribute to the clinical characterization of BVMD. Previous investigations have shown variable patterns of short-wavelength FAF in BVMD, varying from an increased signal, especially visible in the early stages, to a decreased response towards the later stages (Mullins et al. 2005; Wabbels et al. 2006; Boon et al. 2008; Parodi et al. 2016a).

The FAF patterns in BVMD reported by Parodi et al. (2014a) were classified into six types, although no pattern can be considered stage-specific. All of them have been focal and concentrated in the posterior pole. In our study, FAF enabled us to distinguish between BVMD and ARB. Changes in FAF extended beyond the posterior pole in ARB, whereas in BVMD they were focal and limited to the centre of the retina. The hyperfluorescent material gradually disappeared with the progression of RPE cell death and developed into atrophy or scar tissue.

Our results of the FAF patterns in BVMD are consistent with the results obtained by Parodi and coworkers (Parodi et al. 2014a), where the patchy pattern in FAF was the most prevalent. However, Parodi found the patchy pattern across all the stages of BVMD, whereas we found it across the pseudohypopyon, vitelliruptive and atrophic stages. In another study conducted by Parodi (Parodi et al. 2016a), only three stages of BVMD (previtelliform, pseudohypopyon and vitelliruptive) were analysed. Three patterns, that is hypofluorescent, hyperfluorescent and patchy patterns, were observed in 70% of eyes.

FAF is a noninvasive imaging technique that can be used in clinical practice for the diagnosis of BVMD and ARB, as it can visualize more lesions than ophthalmoscopy. The progression of FAF images in BVMD and ARB has not been previously described.

The OCT results in our study are consistent with other studies demonstrating the presence of vitelliform material in the space between RPE and neurosensory retina in the vitelliform and pseudohypopyon stage, as well as the central macular detachment of neurosensory retina in the vitelliform, pseudohypopyon and vitelliruptive stages (Spaide et al. 2006; Querques et al. 2008; Querques et al. 2014). We have found differences in CRT between stages that were close to significance. Schatz and coworkers did not find a significant correlation between CRT and BCVA in Swedish and Danish patients with *BEST1* mutations (Schatz et al. 2010), but correlations between CRT and the stage of the disease have been reported (Querques et al. 2014). In our study, we did not find any significant correlation between CRT and BCVA.

Recently, microperimetry has been used to assess the visual function in patients with BVMD (Parodi et al. 2018). The results indicate that there are relative central scotomata in all stages, including the subclinical stage. The mean retinal sensitivity was reduced in all the BVMD stages; however, the lowest retinal sensitivity was found in the atrophic stage. Moreover, over the last years, preferential hyperacuity perimetry (PHP) (Querques et al. 2011) and chromatic pupilloperimetry (Ben Ner et al. 2019) were assessed in BVMD patients.

The results of SAP testing in ARB patients have not been described so far. We report a general reduction in sensitivity in ARB patients obtained with SAP, although the number of cases is low. This is consistent with the thesis of the presence of an absolute central scotoma in the atrophic stage of BVMD, whereas a relative central scotoma is observed in the other BVMD stages.

The eyes of patients with bestrophinopathies are characteristically hypermetropic, and esotropia is commonly observed (Huysmans 1940; Deutman 1971) and astigmatism and amblyopia are also often found. In our cohort, hypermetropia was also the most common refractive error. Interestingly, the median of hypermetropia decreases at the transition from stage 2 to 3 and 4, possibly indicating a decrease in the elevation of the photoreceptor layer due to rupture, although no statistical significance can be shown. In ARB, hypermetropia and angle closure glaucoma have also been described (Burgess et al. 2008). In our cohort, glaucoma was not a common comorbidity in the ARB and BVMD groups.

To the best of our knowledge, there are only few studies reporting the genotype–phenotype findings with a longitudinal follow-up in the *BEST1* mutations (Parodi et al. 2020). Parodi et al. (2020) focused on structural changes in OCT rather than on the visual function, and their study was a prospective analysis of 21 eyes of 11 patients only in the vitelliform stage examined annually. They found that 62% of the eyes showed an increase in the area of vitelliform deposition. Once the maximal area was reached, progressive flattening of the vitelliform deposition took place, with subsequent flattening of the vitelliform lesion and formation of subretinal fluid. Another study has been recently published by Shah et al. (2020), who analysed a cohort of 36 patients retrospectively. Twenty-two variants in *BEST1* were identified, with three phenotypes distinguished: BVMD, ARB, and adult vitelliform macular dystrophy. They described a ‘beaten metallic retinal appearance extending from the mid periphery to the far periphery’ in the ARB phenotype. Casalino et al. (2020) reported a longitudinal follow-up of 56 eyes from 28 patients with ARB; they detected a significant change in the visual acuity only in patients with a follow-up of more than 5 years and the presence of subretinal fluid and vitelliform material in the majority of subjects that did not substantially change over time. The authors emphasize that ARB is a possible candidate for gene replacement therapy, following the promising results of *BEST1* gene supplementation in the canine model of ARB (Guziewicz et al. 2018).

Despite the limits of its retrospective design, our study provides further knowledge of the variation and natural history of bestrophinopathies through the detailed analysis of many clinical parameters in all the patients from the cohort.

## Supplementary Material

Supplementary Table **Table S1.** Eyes of patients with likely autosomal recessive *BEST1* mutations and phenotype of autosomal recessive bestrophinopathy during the first examination.
**Table S2.** Clinical details of 20 eyes that were found to have progressed during the follow-up.

## Figures and Tables

**Table 1.** Demographic data, family number, nationality, gender, age at the first and last visit, follow-up period in years, number of examinations and stage of Best vitelliform macular dystrophy (BVMD) or presence of autosomal recessive bestrophinopathy (ARB).

Family number	Nationality	Gender	Age at first visit	Age at last visit	Follow-up period (years)	Number of examinations	Stage right eye during first visit	Stage left eye during first visit

MB6-1	German	male	40	51	11	4	5	3
MB6-2	German	Male	45	45	0	1	5	5
MB6-3	German	Male	52	52	0	1	5	5
MB6-4	German	Female	71	76	5	3	5	5
MB19	German	Female	50	50	5	2	3	3
MB27	German	Male	43	57	14	5	5	5
MB28	German	Male	35	35	1	2	2	2
MB31	German	Male	23	37	14	6	2	2
MB38-1	German	Female	22	26	4	2	4	5
MB38-2	German	Female	47	54	7	3	5	5
MB46-1	German	Male	35	35	0	0	4	4
MB46-2	German	Female	48	48	0	1	4	4
MB46-3	German	Female	60	60	0	1	4	4
MB48-1	German	Male	20	25	5	2	2	4
MB48-2	German	Male	29	34	5	2	5	4
MB48-3	German	Female	30	30	0	1	5	4
MB48-4	German	Female	55	60	5	2	5	5
MB70	German	Female	53	58	5	10	2	1
MB72	Italian	Male	36	45	9	5	4	4
MB74	German	Female	28	28	0	1	1	1
MB80	Arabic	Male	35	37	2	2	ARB	ARB
MB83	German	Male	28	37	9	4	5	5
MB88	German	Female	56	56	1	3	2	2
MB86	German	Male	52	53	2	3	ARB	ARB
MB91	German	Male	42	46	4	2	4	4
MB92	German	Male	36	36	0	1	ARB	ARB
MB93	Arabic	Male	16	17	2	3	ARB	ARB
MDS122	German	Female	49	57	8	2	3	5
MDS309	Italian	Male	50	51	1	4	1	1
MDS290	German	Female	44	54	10	7	ARB	ARB
MB64	German	Female	40	44	4	2	3	3

**Table 2.** Spectrum of mutations in the *BEST1* gene in a group of 31 patients affected by BVMD – Best vitelliform macular dystrophy and ARB – autosomal dominant bestrophinopathy.

Family ID and number of patient	Phenotype	Allele 1	Reference (PMID)#	Allele 2	Reference (PMID)	Segregation analysis

Putative dominant cases						
MDS309	BVMD stage 1	c.37C>G;p.R13G	**This study**	–		n.d.
MB72	BVMD stage 4	c.44G>A;p.G15D	20057903	–		yes
MB46 (patients 1–3)	BVMD stage 4	c.72G>T;p.W24C	9700209	–		yes
MB28	BVMD stage 2	c.89A>G;p.K30R	10798642	–		n.d.
MB6 (patients 1–4)	BVMD 7 eyes stage 5, 1 eye stage 3	c.652C>T;p.R218C	9700209	–		yes
MB48 (patients 1–4)	BVMD 4 eyes stage 5, 3 eyes stage 4	c.703G>T;p.V235L	11241846	–		yes
MB88	BVMD stage 2	c.728C>T;p.A243V	10737974	–		n.d.
MB31	BVMD stage 2	c.728C>T;p.A243V	10737974	–		n.d.
MB27	BVMD stage 5	c.728C>T;p.A243V	10737974	–		n.d.
MDS122	BVMD 1 eye stage 3, 1 eye stage 5	c.884T>C;p.I295T	12187431	–		yes
MB38 (patients 1 + 2)	BVMD 3 eyes stage 5, 1 eye stage 4	c.884T>C;p.I295T	12187431	–		yes
MB19	BVMD stage 3	c.884_886del; p.I295del	9700209	–		yes
MB86	BVMD stage 5	c.884_886del; p.I295del	9700209	–		n.d.
MB91	BVMD stage 4	c.884_886del; p.l295del	9700209	–		n.d.
MB74	BVMD stage 1	c.932T>G;p.V311G	10737974	–		n.d.
Putative recessive cases						
MB92	ARB	c.397A>C; p.N133H	21273940	c.712del; p.Q238Rfs*3	**this study**	yes
MB80	ARB	c.400C>G;p.L134V	17287362	c.400C>G;p.L134V	17287362	yes
MB83	ARB	c.422G>A; p.R141H	10854112	c.422G>A; p.R141H	10854112	n.d.
MB64	BVMD stage 3	c.584C>T;p.A195V	10798642	c.934G>A; p.D312N	10854112	n.d.
MDS290	ARB	c.779del; p.P260Qfs*29	14517959	c.779del; p.P260Qfs*29	14517959	n.d.
MB93	ARB	c.889C>T;p.P297S	10453731	c.1315C>T; p.Q439*	**this study**	n.d.
Unclear inheritance						
MB70	BVMD 1 eye stage 1, 1 eye stage 2	c.422G>A; p.R141H	10854112			n.d.

# If multiple references are known, only the first description is given.

Other abbreviations: PMID – PubMed Unique Identifier, n.d. – not done.

**Table 3.** Gass fundus classification as well as optical coherence tomography (OCT) representative cases (vertical line) and fundus autofluorescence (FAF) patterns (horizontal line) of 52 eyes with Best vitelliform macular dystrophy (BVMD) and ten eyes with autosomal recessive bestrophinopathy (ARB).

	Parodi FAF pattern							
Gass fundus classification and OCT profile	Normal 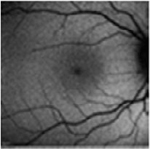	Hyperfluorescent 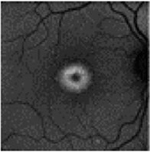	Multifocal 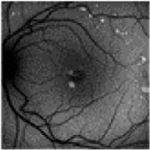	Patchy 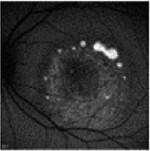	Hypofluorescent 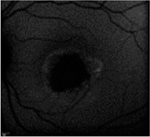	Spoke-like 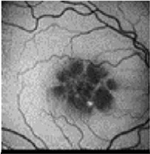	Diffuse 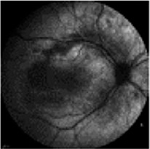	Total

Previtelliform (Stage 1) 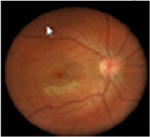 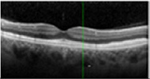	2	0	3	0	0	0	0	5 (8%)
Vitelliform (Stage 2) 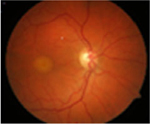 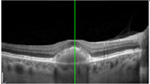	1	7	0	0	0	0	0	8 (12%)
Pseudohypopyon (Stage 3) 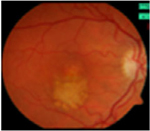 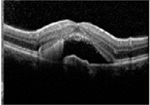	0	3	0	3	0	0	0	6 (10%)
Vitelliruptive (Stage 4) 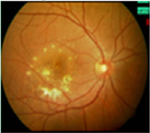 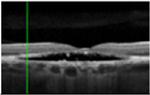	0	0	0	14	0	0	0	14 (23%)
Atrophic (Stage 5) 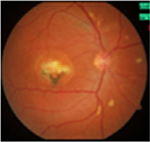 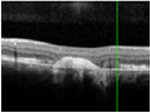	0	0	2	8	7	2	0	19 (31%)
Autosomal recessive bestrophinopathy 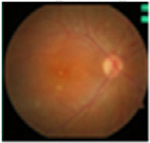 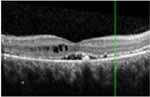	0	0	0	0	0	0	10	10 (16%)
Total	3 (5%)	10 (16%)	5 (8%)	25 (40%)	7 (12%)	2 (3%)	10 (16%)	62 (100%)

## References

[R1] Arden GB & Constable PA (2006): The electro-oculogram. Prog Retin Eye Res 25: 207–248. 16473035 10.1016/j.preteyeres.2005.11.001

[R2] Bakall B, Radu RA, Stanton JB (2007): Enhanced accumulation of A2E in individuals homozygous or heterozygous for mutations in *BEST1* (VMD2). Exp Eye Res 85: 34–43. 17477921 10.1016/j.exer.2007.02.018

[R3] Ben Ner D, Sher I, Hamburg A (2019): Chromatic pupilloperimetry for objective diagnosis of Best vitelliform macular dystrophy. Clin Ophthalmol 13: 465–475. 30880907 10.2147/OPTH.S191486 PMC6407903

[R4] Birtel J, Gliem M, Herrmann P, MacLaren RE, Bolz HJ & Charbel Issa P (2020): Peripapillary sparing in autosomal recessive bestrophinopathy. Ophthalmol Retina 4: 523–529. 32147488 10.1016/j.oret.2019.12.008

[R5] Bitner H, Mizrahi-Meissonnier L, Griefner G, Erdinest I, Sharon D & Banin E (2011): A homozygous frameshift mutation in *BEST1* causes the classical form of Best disease in an autosomal recessive mode. Investig Ophthalmol Vis Sci 52: 5332–5338. 21467170 10.1167/iovs.11-7174

[R6] Bitner H, Schatz P, Mizrahi-Meissonnier L, Sharon D & Rosenberg T (2012): Frequency, genotype, and clinical spectrum of best vitelliform macular dystrophy: data from a national center in Denmark. Am J Ophthalmol 154: 403–412.e4. 22633354 10.1016/j.ajo.2012.02.036

[R7] Boon CJ, Jeroen Klevering B, Keunen JE, Hoyng CB & Theelen T (2008): Fundus autofluorescence imaging of retinal dystrophies. Vision Res 48: 2569–2577. 18289629 10.1016/j.visres.2008.01.010

[R8] Boon CJ, Klevering BJ, Leroy BP, Hoyng CB, Keunen JE & den Hollander AI (2009): The spectrum of ocular phenotypes caused by mutations in the *BEST1* gene. Prog Retin Eye Res 28: 187–205. 19375515 10.1016/j.preteyeres.2009.04.002

[R9] Boon CJ, van den Born LI, Visser L (2013): Autosomal recessive bestrophinopathy: differential diagnosis and treatment options. Ophthalmology 120: 809–820. 23290749 10.1016/j.ophtha.2012.09.057

[R10] Burgess R, Millar ID, Leroy BP (2008): Biallelic mutation of *BEST1* causes a distinct retinopathy in humans. Am J Hum Genet 82: 19–31. 18179881 10.1016/j.ajhg.2007.08.004 PMC2253971

[R11] Casalino G, Khan KN, Armengol M (2020): Autosomal recessive bestrophinopathy: clinical features, natural history and genetic findings in preparation for clinical trials. Ophthalmology 128: 706–718. 33039401 10.1016/j.ophtha.2020.10.006 PMC8062850

[R12] Deutman AF (1971): The hereditary dystrophies of the posterior pole of the eye. Springfield: Charles C. Thomas, pp. 198–299.

[R13] Fokkema IF, den Dunnen JT & Lovd TPE (2005): Easy creation of a locus-specific sequence variation database using an “LSDB-in-a-box” approach. Hum Mutat 26: 63–68. 15977173 10.1002/humu.20201

[R14] Frangieh GT, Green WR & Fine SL (1982): A histopathologic study of Best’s macular dystrophy. Arch Ophthalmol 100: 1115–1121. 7092655 10.1001/archopht.1982.01030040093017

[R15] Gao FJ, Qi YH, Hu FY (2020): Mutation spectrum of the bestrophin-1 gene in a large Chinese cohort with bestrophinopathy. Br J Ophthalmol 104: 846–851. 31519547 10.1136/bjophthalmol-2019-314679

[R16] Gao T, Tian C, Hu Q, Liu Z, Zou J, Huang L & Zhao M (2018): Clinical and mutation analysis of patients with best vitelliform macular dystrophy or autosomal recessive bestrophinopathy in Chinese population. Biomed Res Int 18: 4582816. 10.1155/2018/4582816 PMC6220750 30498755

[R17] Gass JDM (1997): Best’s disease. In: Stereoscopic atlas of macular disease. Diagnosis and treatment. St. Louis, London, Philadelphia, Sydney, Toronto: Mosby 304–313.

[R18] Guziewicz KE, Cideciyan AV, Beltran WA (2018): *BEST1* gene therapy corrects a diffuse retina-wide microdetachment modulated by light exposure. Proc Natl Acad Sci USA 115: E2839–E2848. 29507198 10.1073/pnas.1720662115 PMC5866594

[R19] Hartzell HC, Qu Z, Yu K, Xiao Q & Chien LT (2008): Molecular physiology of bestrophins: multifunctional membrane proteins linked to best disease and other retinopathies. Physiol Rev 88: 639–672. 18391176 10.1152/physrev.00022.2007

[R20] Huysmans J (1940): Exudative central detachment of the retina in a family (macular pseudo-cysts).Ophthalmologica 99:449–455.

[R21] Kinnick TR, Mullins RF, Dev S (2011): Autosomal recessive vitelliform macular dystrophy in a large cohort of vitelliform macular dystrophy patients. Retina 31: 581–595. 21273940 10.1097/IAE.0b013e318203ee60

[R22] Kräamer F, White K, Pauleikhoff D (2000): Mutations in the VMD2 gene are associated with juvenile-onset vitelliform macular dystrophy (Best disease) and adult vitelliform macular dystrophy but not age-related macular degeneration. Eur J Hum Genet 8: 286–292. 10854112 10.1038/sj.ejhg.5200447

[R23] Lima de Carvalho JR Jr, Paavo M, Chen L, Chiang J, Tsang SH & Sparrow JR (2019): Multimodal imaging in best vitelliform macular dystrophy. Invest Ophthalmol Vis Sci 60: 2012–2022. 31070670 10.1167/iovs.19-26571 PMC6735800

[R24] Marmorstein AD, Cross HE & Peachey NS (2009): Functional roles of bestrophins in ocular epithelia. Prog Retin Eye Res 28: 206–226. 19398034 10.1016/j.preteyeres.2009.04.004 PMC2740978

[R25] Marmorstein AD, Marmorstein LY, Rayborn M, Wang X, Hollyfield JG & Petrukhin K (2000): Bestrophin, the product of the Best vitelliform macular dystrophy gene (VMD2), localizes to the basolateral plasma membrane of the retinal pigment epithelium. Proc Natl Acad Sci USA 97: 12758–12763. 11050159 10.1073/pnas.220402097 PMC18837

[R26] McCulloch DL, Marmor MF, Brigell MG, Hamilton R, Holder GE, Tzekov R & Bach M (2015): ISCEV Standard for full-field clinical electroretinography (2015 update). Doc Ophthalmol 130: 1–12. 10.1007/s10633-014-9473-7 25502644

[R27] Milenkovic A, Milenkovic VM, Wetzel CH & Weber BHF (2018): *BEST1* protein stability and degradation pathways differ between autosomal dominant best disease and autosomal recessive bestrophinopathy accounting for the distinct retinal phenotypes. Hum Mol Genet 27: 1630–1641. 29668979 10.1093/hmg/ddy070 PMC5905664

[R28] Miller SA (1978): Fluorescence in Best’s vitelliform dystrophy, lipofuscin, and fundus flavimaculatus. Br J Ophthalmol 62: 256–260. 646985 10.1136/bjo.62.4.256 PMC1043198

[R29] Mullins RF, Oh KT, Heffron E, Hageman GS & Stone EM (2005): Late development of vitelliform lesions and flecks in a patient with best disease: clinicopathologic correlation. Arch Ophthalmol 123: 1588–1594. 16286623 10.1001/archopht.123.11.1588

[R30] Parodi MB, Castellino N, Iacono P, Chowers I, Empeslidis T, Goldstein M & Bandello F (2018): Microperimetry in Vitelliform macular dystrophy. Retina 38: 841–848. 28301340 10.1097/IAE.0000000000001600

[R31] Parodi MB, Iacono P, Campa C, Del Turco C & Bandello F (2014a): Fundus autofluorescence patterns in Best vitelliform macular dystrophy. Am J Ophthalmol 158: 1086–1092. 25068640 10.1016/j.ajo.2014.07.026

[R32] Parodi MB, Iacono P, Del Turco C, Triolo G & Bandello F (2016a): Functional assessment of the fundus autofluorescence pattern in Best vitelliform macular dystrophy. Graefes Arch Clin Exp Ophthalmol 254: 1297–1302. 26490373 10.1007/s00417-015-3194-9

[R33] Parodi MB, Romano F, Arrigo A, Di Nunzio C, Buzzotta A, Alto G & Bandello F (2020): Natural course of the vitelliform stage in best vitelliform macular dystrophy: a fiveyear follow-up study. Graefes Arch Clin Exp Ophthalmol 258: 297–301. 31848692 10.1007/s00417-019-04454-4

[R34] Parodi MB, Sacconi R, Iacono P, Del Turco C & Bandello F (2016b): Choroidal thickness in Best vitelliform macular dystrophy. Retina 36: 764–769. 26447398 10.1097/IAE.0000000000000759

[R35] Parodi MB, Zucchiatti I, Fasce F & Bandello F (2014b): Bilateral choroidal excavation in best vitelliform macular dystrophy. Ophthalmic Surg Lasers Imaging. Retina 14: e8–e10. 10.3928/23258160-20140205-01 24512759

[R36] Pasquay C, Wang LF, Lorenz B & Preising MN (2015): Bestrophin 1–phenotypes and functional aspects in bestrophinopathies. Ophthalmic Genet 36: 193–212. 24328569 10.3109/13816810.2013.863945

[R37] Piñeiro-Gallego T, Álvarez M, Pereiro I, Campos S, Sharon D, Schatz P & Valverde D (2011): Clinical evaluation of two consanguineous families with homozygous mutations in *BEST1*. Mol Vis 17: 1607–1617. 21738390 PMC3123162

[R38] Querques G, Atmani K, Bouzitou-Mfoumou R, Leveziel N, Massamba N & Souied EH (2011): Preferential hyperacuity perimeter in best vitelliform macular dystrophy. Retina 31: 959–966. 21242858 10.1097/IAE.0b013e3181f441c1

[R39] Querques G, Regenbogen M, Quijano C, Delphin N, Soubrane G & Souied EH (2008): High-definition optical coherence tomography features in vitelliform macular dystrophy. Am J Ophthalmol 146: 501–507. 18619572 10.1016/j.ajo.2008.05.029

[R40] Querques G, Zerbib J, Georges A (2014): Multimodal analysis of the progression of Best vitelliform macular dystrophy. Mol Vis 27: 575–592. PMC4000718 24791142

[R41] Schatz P, Bitner H, Sander B, Holfort S, Andreasson S, Larsen M & Sharon D (2010): Evaluation of macular structure and function by OCT and electrophysiology in patients with vitelliform macular dystrophy due to mutations in *BEST1*. Invest Ophthalmol Vis Sci 51: 4754–4765. 20375334 10.1167/iovs.10-5152

[R42] Shah M, Broadgate S, Shanks M (2020): Association of clinical and genetic heterogeneity with *BEST1* sequence variations. JAMA Ophthalmol 2: e200666. 10.1001/jamaophthalmol.2020.0666 PMC7118667 32239196

[R43] Sodi A, Passerini I, Murro V, Caputo R, Bacci GM, Bodoj M, Torricelli F & Menchini U (2012): *BEST1* sequence Variants in Italian patients with vitelliform macular dystrophy. Mol Vis 18: 2736–2748. 23213274 PMC3513188

[R44] Spaide RF, Noble K, Morgan A & Freund KB (2006): Vitelliform macular dystrophy. Ophthalmology 113: 1392–1400. 16877078 10.1016/j.ophtha.2006.03.023

[R45] Tian L, Sun T, Xu K, Zhang X, Peng X & Li Y (2017): Screening of *BEST1* gene in a Chinese cohort with Best vitelliform macular dystrophy or autosomal recessive bestrophinopathy. Invest Ophthalmol Vis Sci 58: 3366–3375. 28687848 10.1167/iovs.17-21999

[R46] Wabbels B, Preising MN, Kretschmann U, Demmler A & Lorenz B (2006): Genotype-phenotype correlation and longitudinal course in ten families with Best vitelliform macular dystrophy. Graefes Arch Clin Exp Ophthalmol 244: 1453–1466. 16612637 10.1007/s00417-006-0286-6

[R47] Weisschuh N, Obermaier CD, Battke F (2020): Genetic architecture of inherited retinal degeneration in Germany: A large cohort study from a single diagnostic center over a 9-year period. Hum Mutat 41: 1514–1527. 32531858 10.1002/humu.24064

