# Supplementary material for: Disease expression caused by different variants in the BEST1 gene: genotype and phenotype findings in bestrophinopathies
Source: Acta Ophthalmol. Author manuscript; Available in PMC 2022 Jul 27. (PMC9328113; doi:10.1111/aos.14958)
Supplement: Supplementary Table — Table S1. Eyes of patients with likely autosomal recessive BEST1 mutations and phenotype of autosomal recessive bestrophinopathy during the first examination. Table S2. Clinical details of 20 eyes that were found to have progressed during the follow-up. [file NIHMS1794562-supplement-Supplementary_Table.docx]

Supplemental Table 1. Eyes of patients with likely autosomal recessive *BEST1* mutations and phenotype of autosomal recessive bestrophinopathy during the first examination. Abbreviations: ARB - autosomal recessive bestrophinopathy, BVMD - Best vitelliform macular dystrophy, OCT - optical coherence tomography; RE - right eye; LE - left eye; n.d.-no data.

| Patient ID | *BEST1* genotype  Phenotype | Fundus RE | Fundus LE | FAF RE | FAF LE | OCT RE | OCT LE |
| --- | --- | --- | --- | --- | --- | --- | --- |
| MB80 | c.400C>G;p.L134V /c.400C>G;p.L134V  ARB | 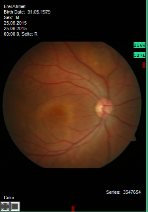 | 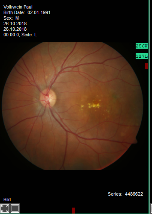 | 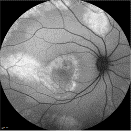 | 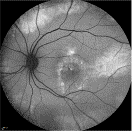 | 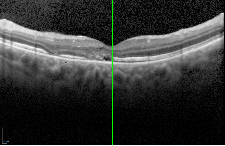 | 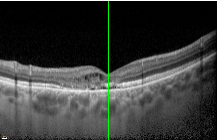 |
| MB92 | c.397A>C;p.N133H /c.712del;p.Q238Rfs*3  RB | 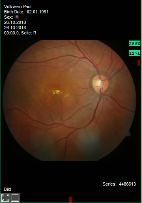 | 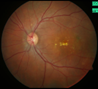 | 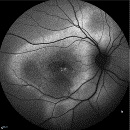 | 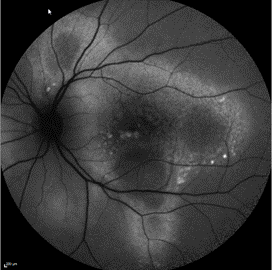 | 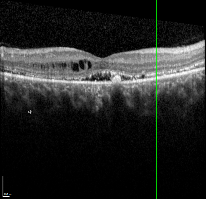 | 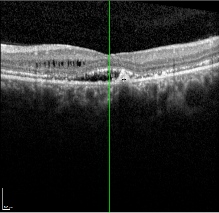 |
| MB93 | c.889C>T;p.P297S/ c.1315C>T;p.Q439*  RB | n.d. | n.d. | 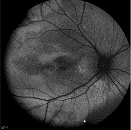 | 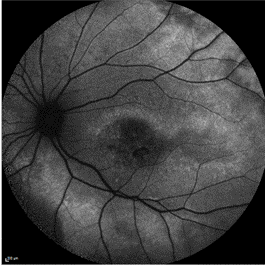 | 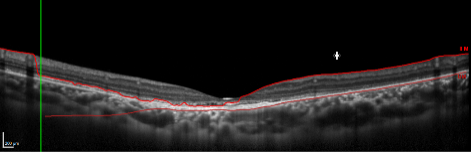 | 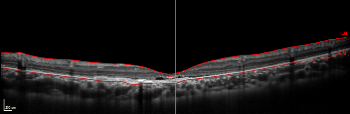 |
| MDS290 | c.779del;p.P260Qfs*29 p/ c.779del;p.P260Qfs*29  ARB | 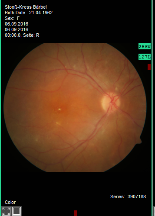 | 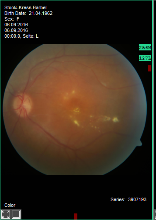 | 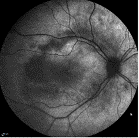 | 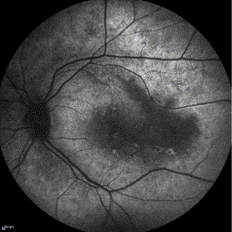 | 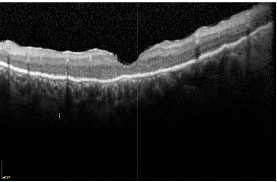 | 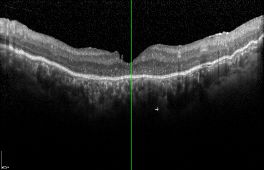 |
| MB86 | c.884_886delITCAp.ILe295del | 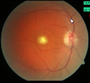 | 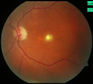 | 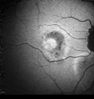 | 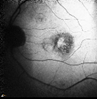 | 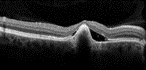 | 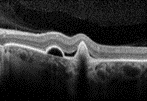 |

Supplemental Table 2. Clinical details of 20 eyes that were found to have progressed during the follow-up. Abbreviations: BCVA – best-corrected visual acuity; FAF –

fundus autofluorescence; OCT - optical coherence tomography; RE - right eye; LE - left eye; n.d. – no data).

| Patient ID | Phenotype, mode of inheritance, eye | Follow-up period (years) | BCVA at baseline | BCVA at the end of the follow-up | Fundus at baseline | Fundus at the end of the follow-up | FAF at baseline | FAF at the end of the follow-up | OCT at baseline | OCT at the end of the follow-up |
| --- | --- | --- | --- | --- | --- | --- | --- | --- | --- | --- |
| MB70 | BVMD, unclear, RE | 5 | 1.0  (stage 1) | 0.6  (stage 2) | 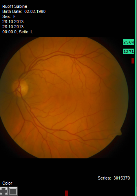 | 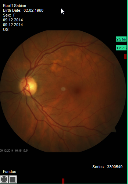 | 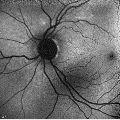 | 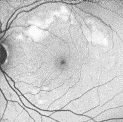 | 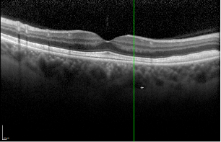 | 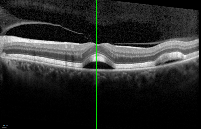 |
| MB70 | BVMD, unclear, LE | 5 | 0.8  (stage 2) | 0.3  (stage 3) | 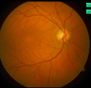 | 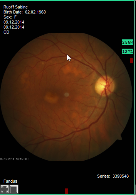 | 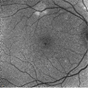 | 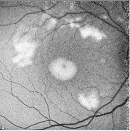 | 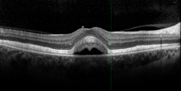 | 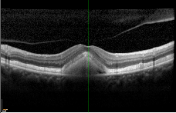 |
| MB88 | BVMD, dominant, RE | 2 | 0.8  (stage 2) | 0.1  (stage 5) | 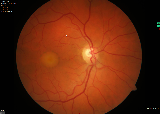 | 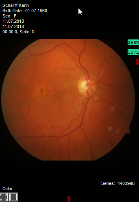 | 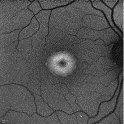 | 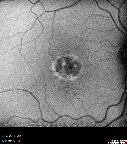 | 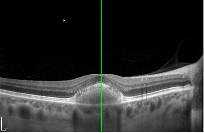 | 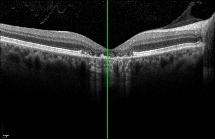 |
| MB31 | BVMD, dominant, RE | 14 | 0.8 (stage 2) | 0.5 (stage 2) | 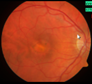 | 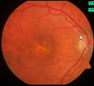 | 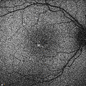 | 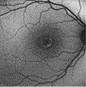 | 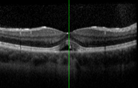 | 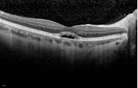 |
| MB31 | BVMD, dominant, LE | 14 | 0.8 (stage 2) | 0.4 (stage 2) | 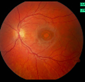 | 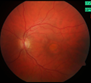 | 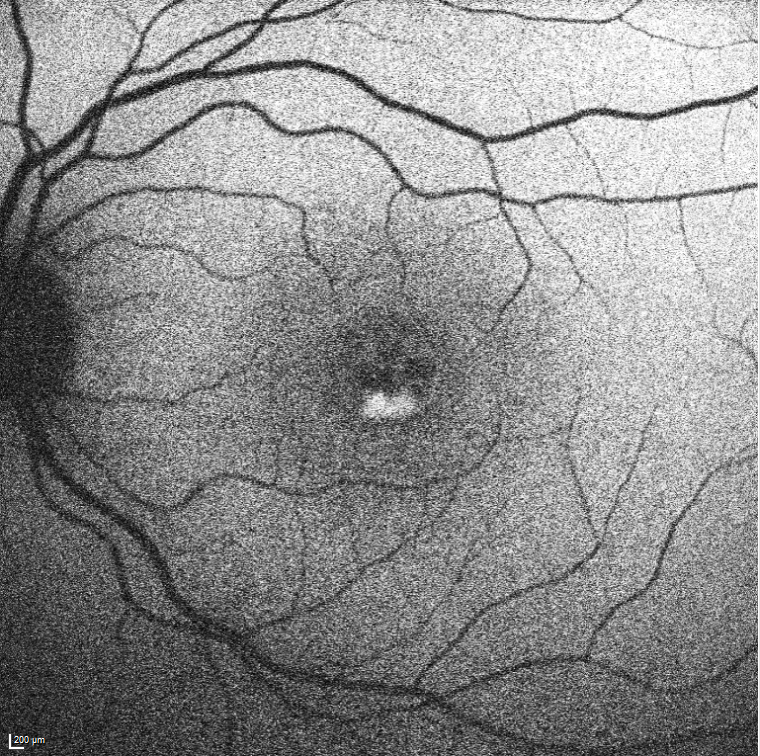 | 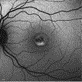 | 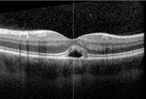 | 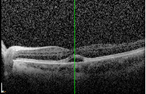 |
| MB19 | BVMD, dominant, RE | 15 | 1.0  (stage 3) | 0.4  (stage 5) | 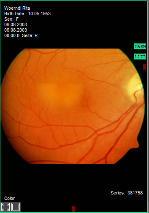 | n.d. | \| n.d. \| \| --- \| | 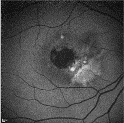 | \| n.d. \| \| --- \| | 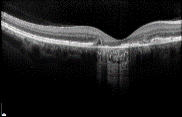 |
| MB19 | BVMD, dominant, LE | 15 | 1.0  (stage 3) | 0.7  (stage 4) | 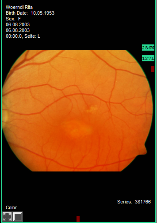 | 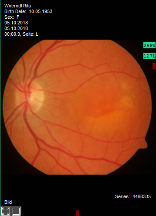 | \| n.d. \| \| --- \| | 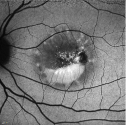 | \| n.d. \| \| --- \| | 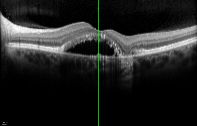 |
| MDS122 | BVMD, dominant, RE | 8 | 0.6  (stage 3) | 0.5  (stage 5) | 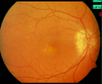 | 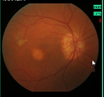 | 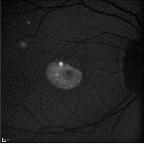 | 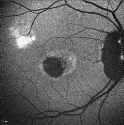 | \| n.d. \| \| --- \| | \| n.d. \| \| --- \| |
| MB6 – member 1 | BVMD, dominant, RE | 11 | 0.8  (stage 3) | 0.3  (stage 4) | 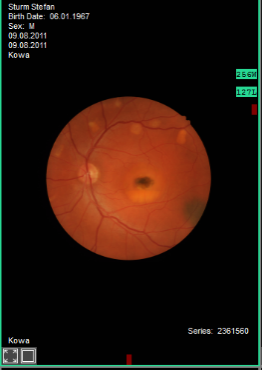 | 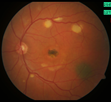 | 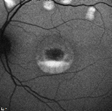 | 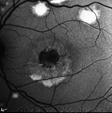 | 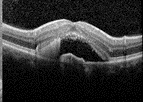 | 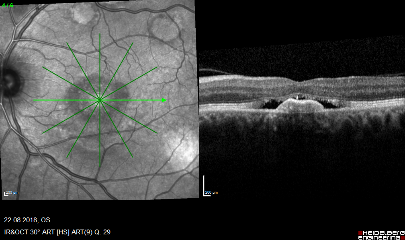 |
| MB72 | BVMD, dominant, RE | 9 | 1.0  (stage 4) | 0.5  (stage 4) | 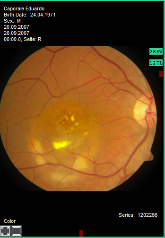 | 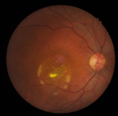 | 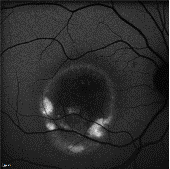 | 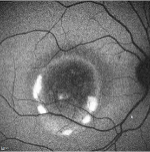 | 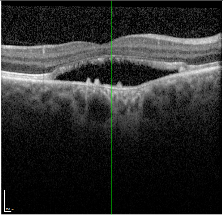 | 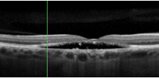 |
| MB72 | BVMD, dominant, LE | 9 | 1.0  (stage 4) | 0.8  (stage 4) | 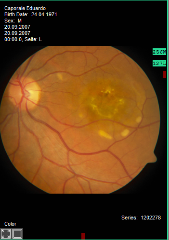 | 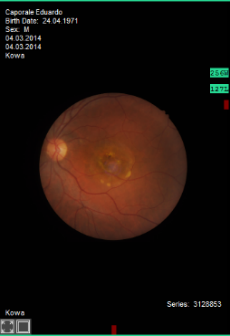 | 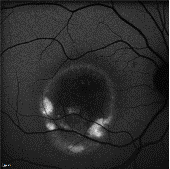 | 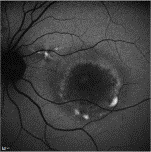 | 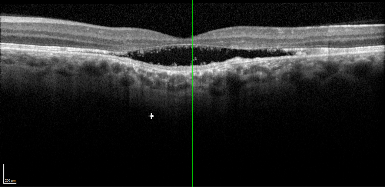 | 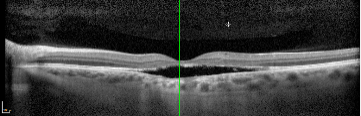 |
| MB83 | BVMD, dominant, RE | 9 | 0.3 (stage 4) | 0.1 (stage 5) | 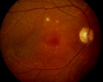 | 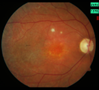 | 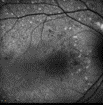 | 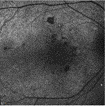 | 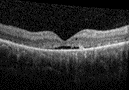 | 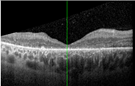 |
| MB6 -member 4 | BVMD, dominant, LE | 5 | 0.7 (stage 5) | 0.5 (stage 5) | n.d. | 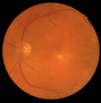 | 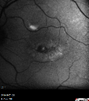 | 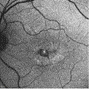 | n.d. | 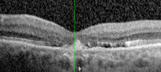 |
| MB 27 | BVMD, dominant, RE | 14 | 0.6 (stage 5) | 0.125 (stage 5) | 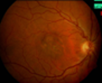 | 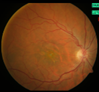 | 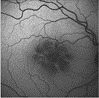 |  |  |  |
| MB 27 | BVMD, dominant, LE | 14 | 0.5 (stage 5) | 0.16 (stage 5) |  |  |  |  |  |  |
| MB48 (member 2) | BVMD, dominant, RE | 5 | 0.3 (stage 5) | 0.2 (stage 5) |  |  |  |  |  |  |
| MB93 | ARB, recessive, RA | 2 | 0.4 | 0.25 | n.d. | n.d. | \| n.d. \| \| --- \| |  | \| n.d. \| \| --- \| |  |
| MB93 | ARB, recessive , LE | 2 | 0.4 | 0.3 | n.d. | n.d. | \| n.d. \| \| --- \| |  | \| n.d. \| \| --- \| |  |
| MB 86 | ARB, recessive, RA | 2 | 0.2 | 0.1 |  |  |  |  |  |  |
| MB 86 | ARB, recessive , LE | 2 | 0.1 | 0.05 |  |  |  |  |  |  |
